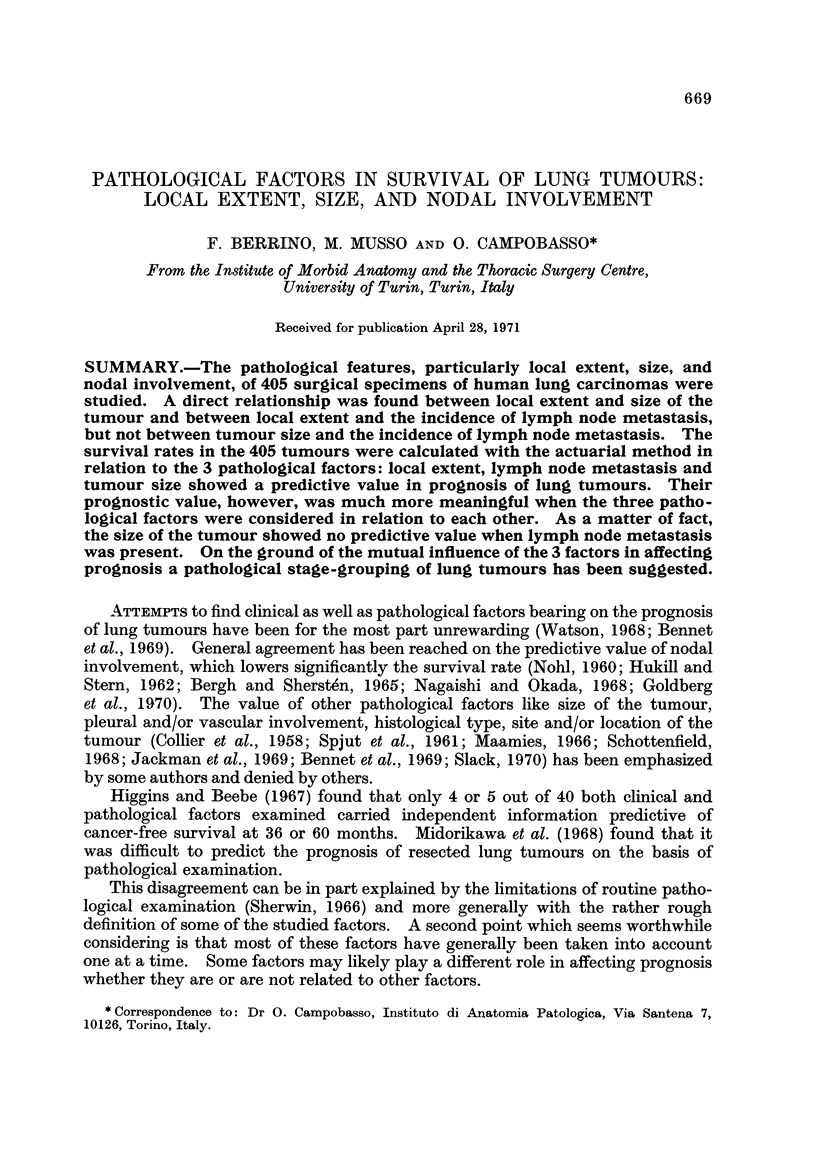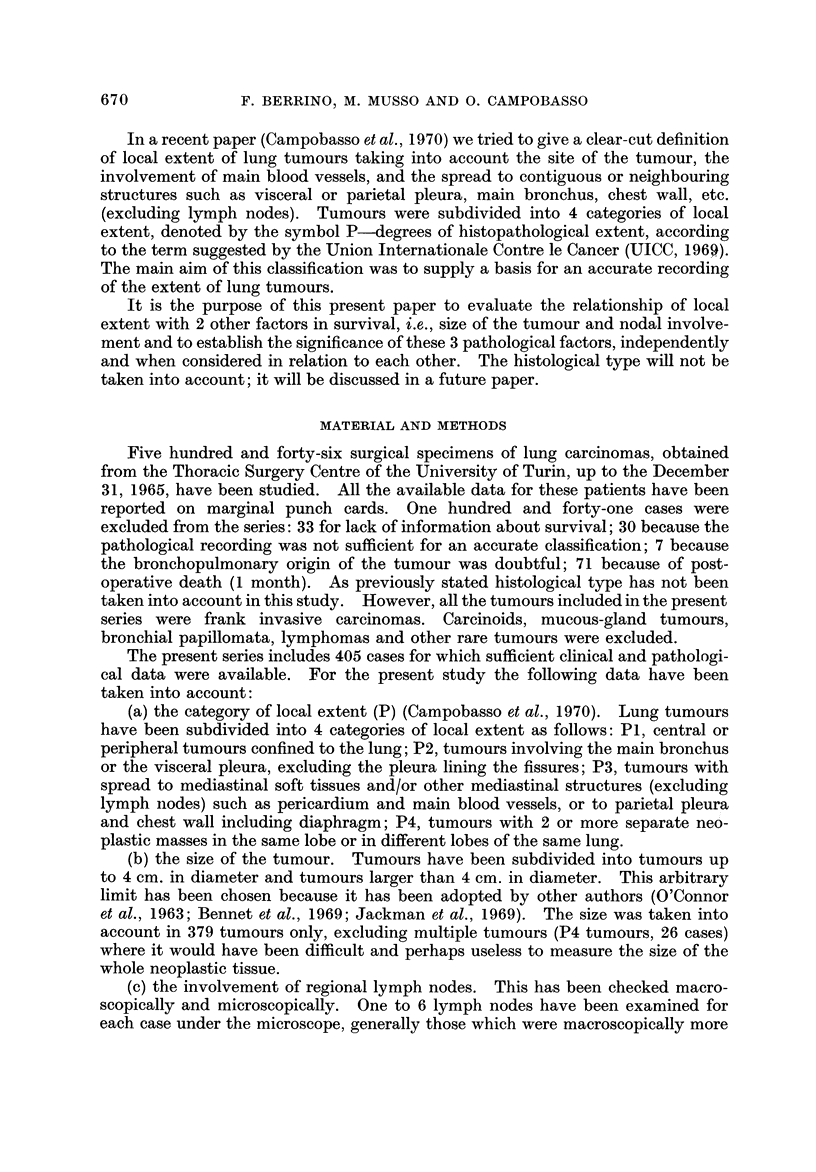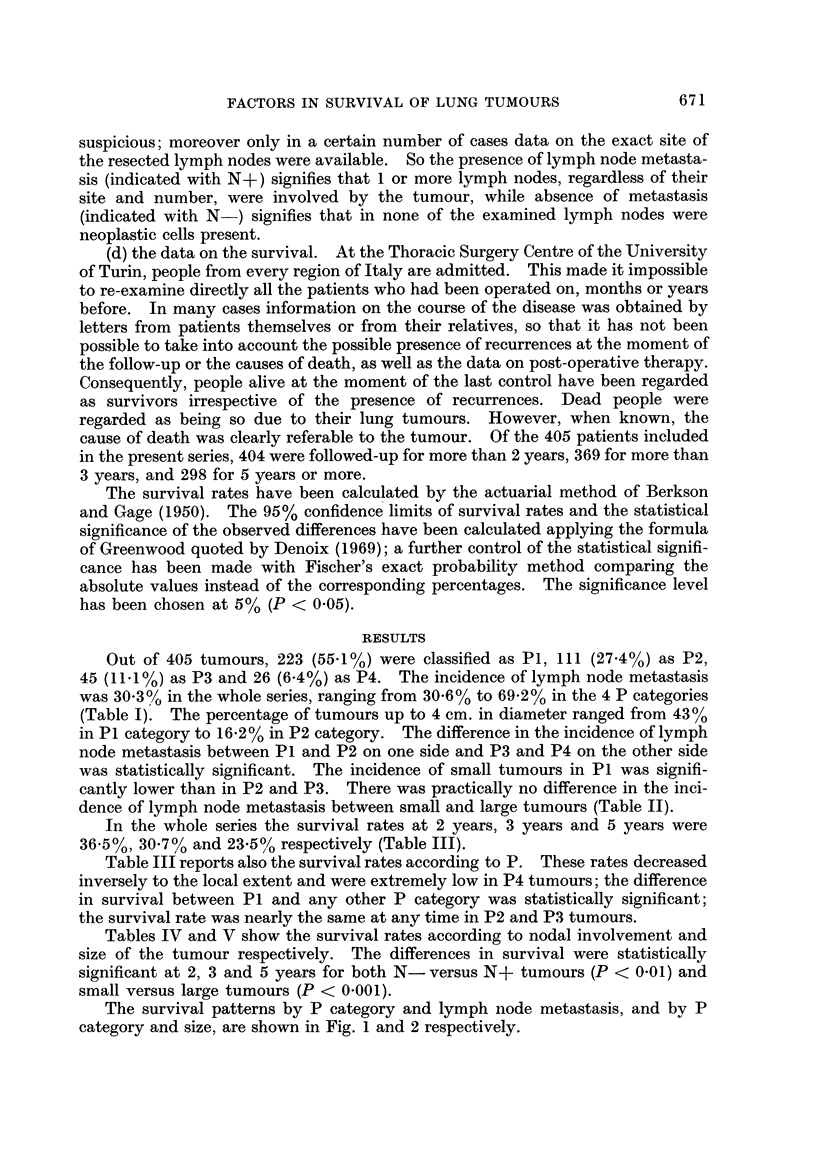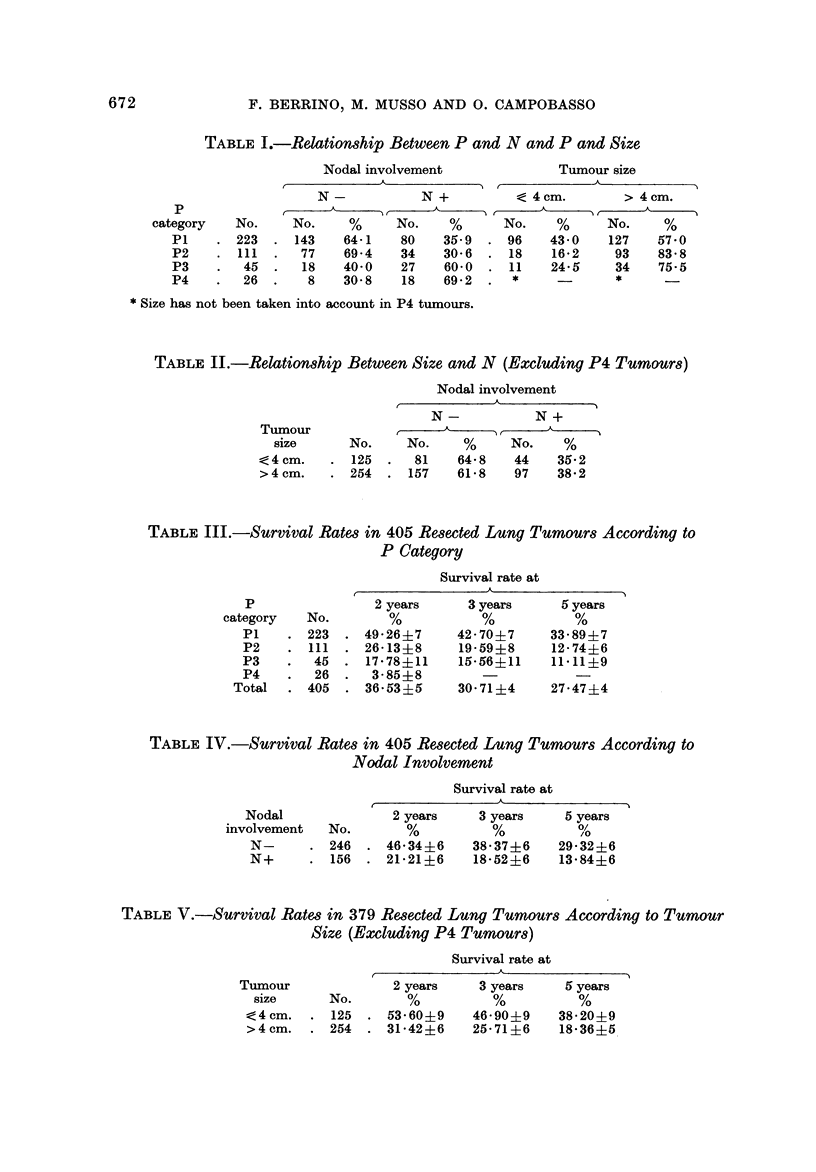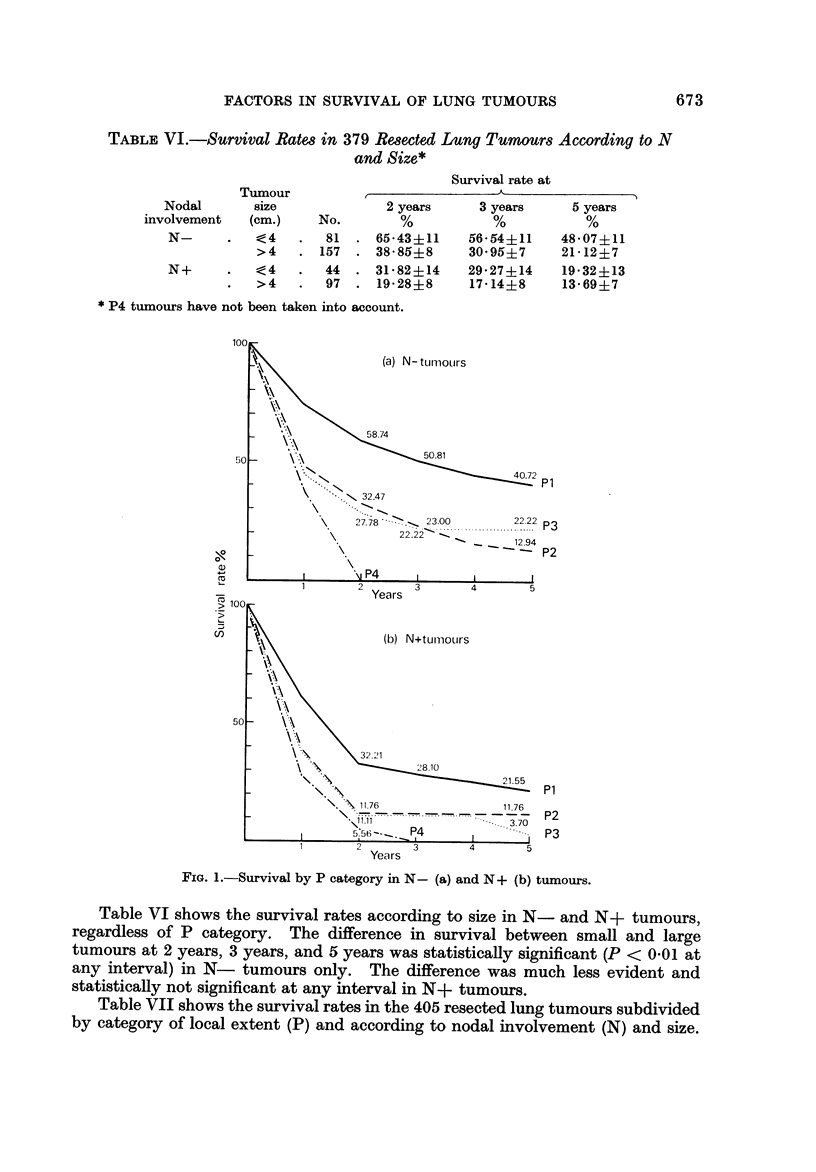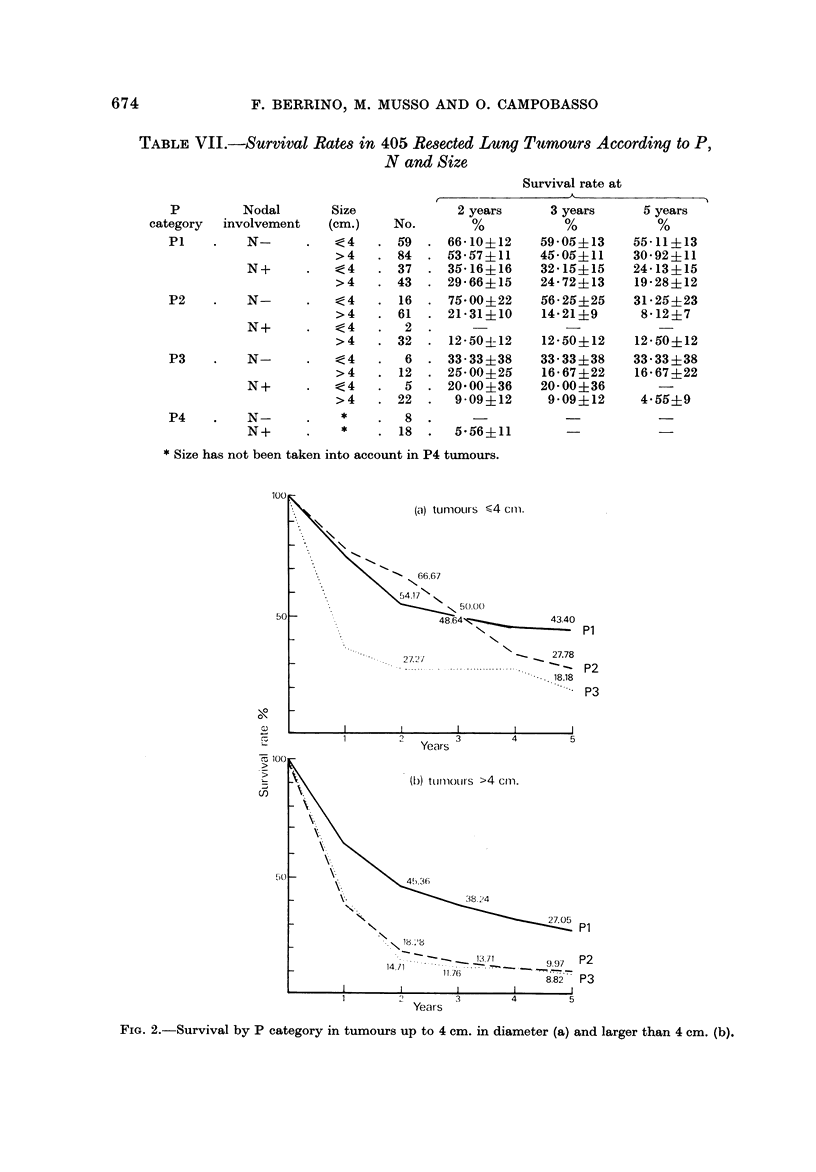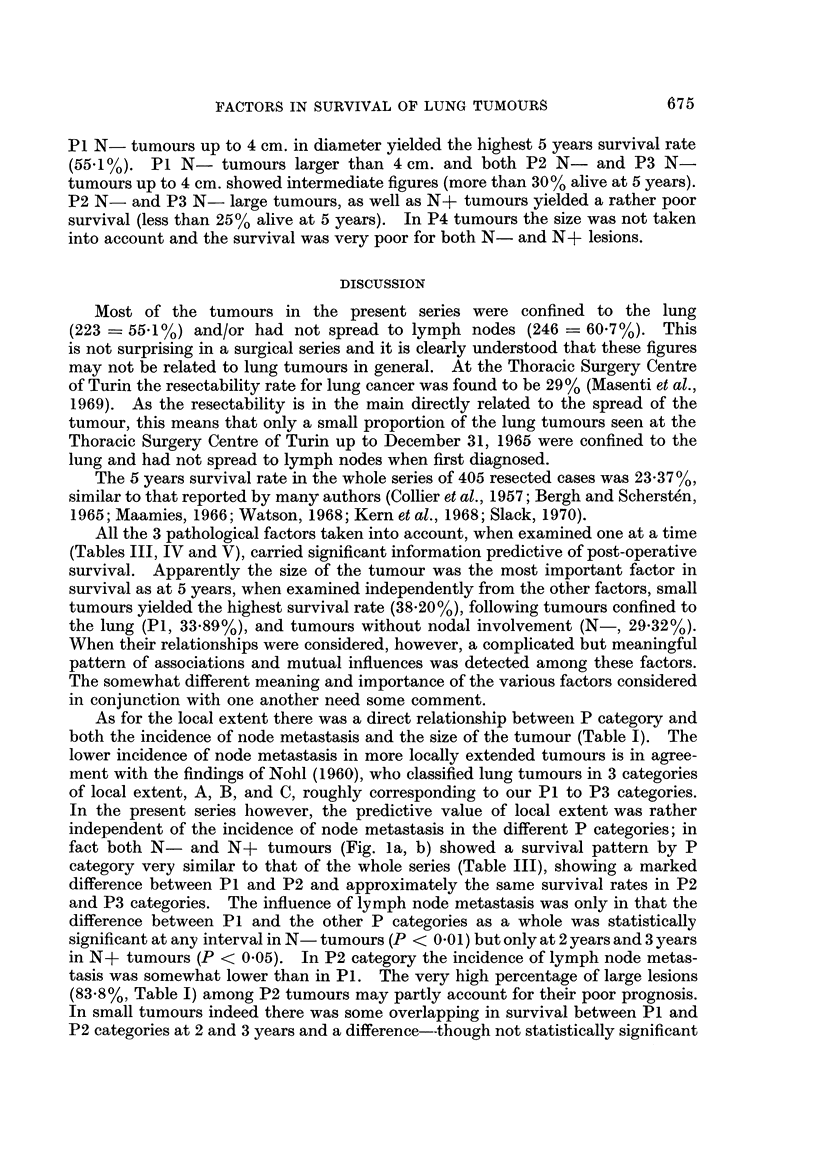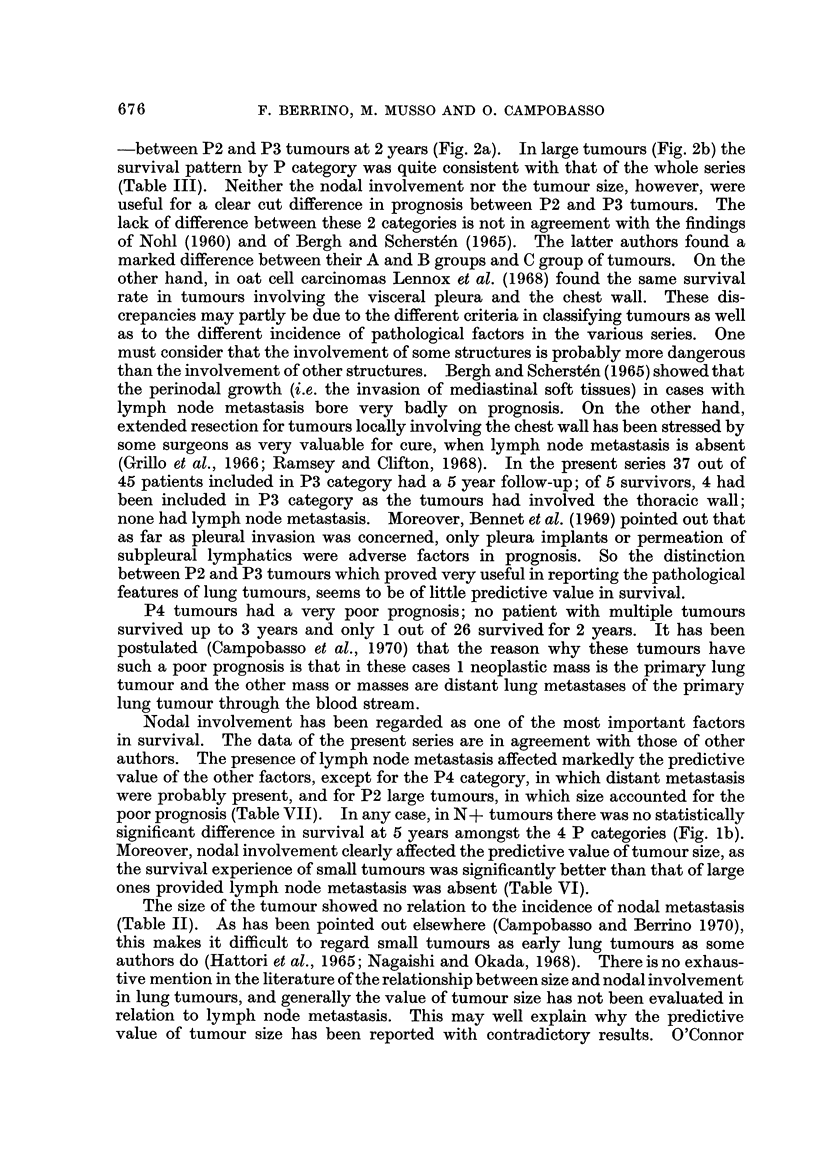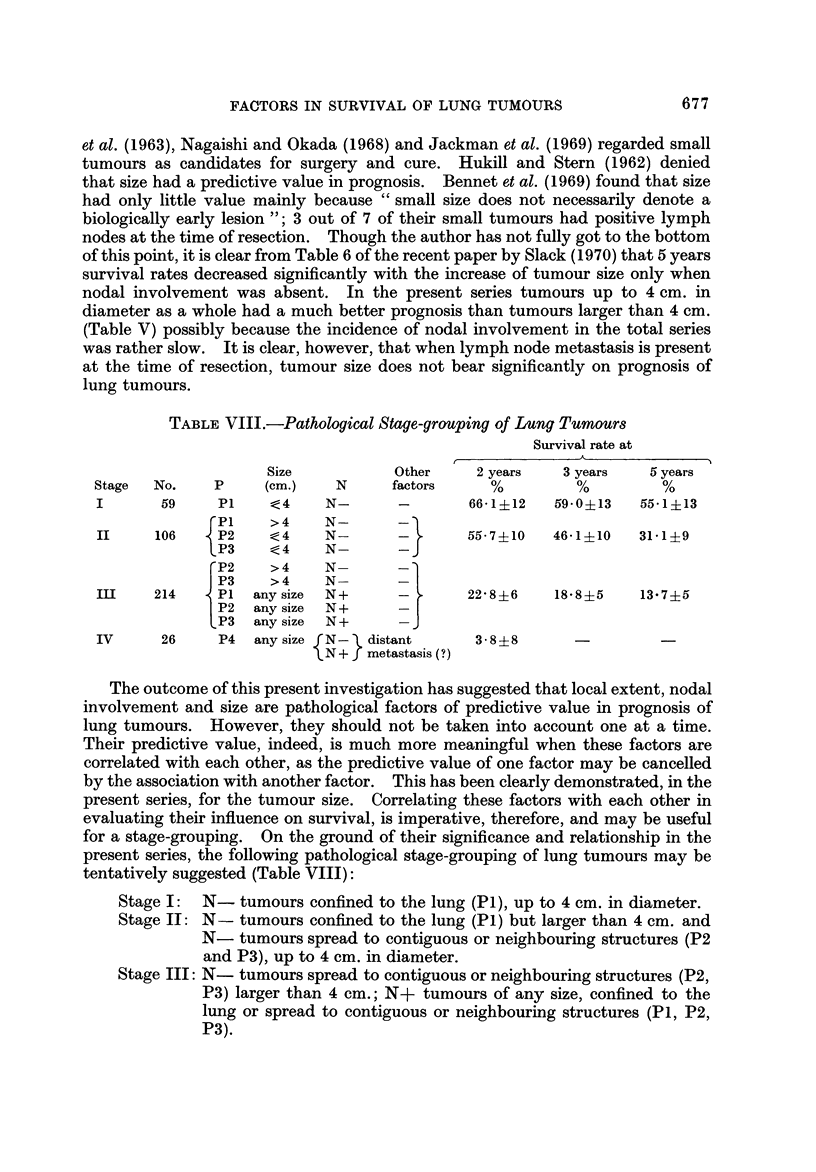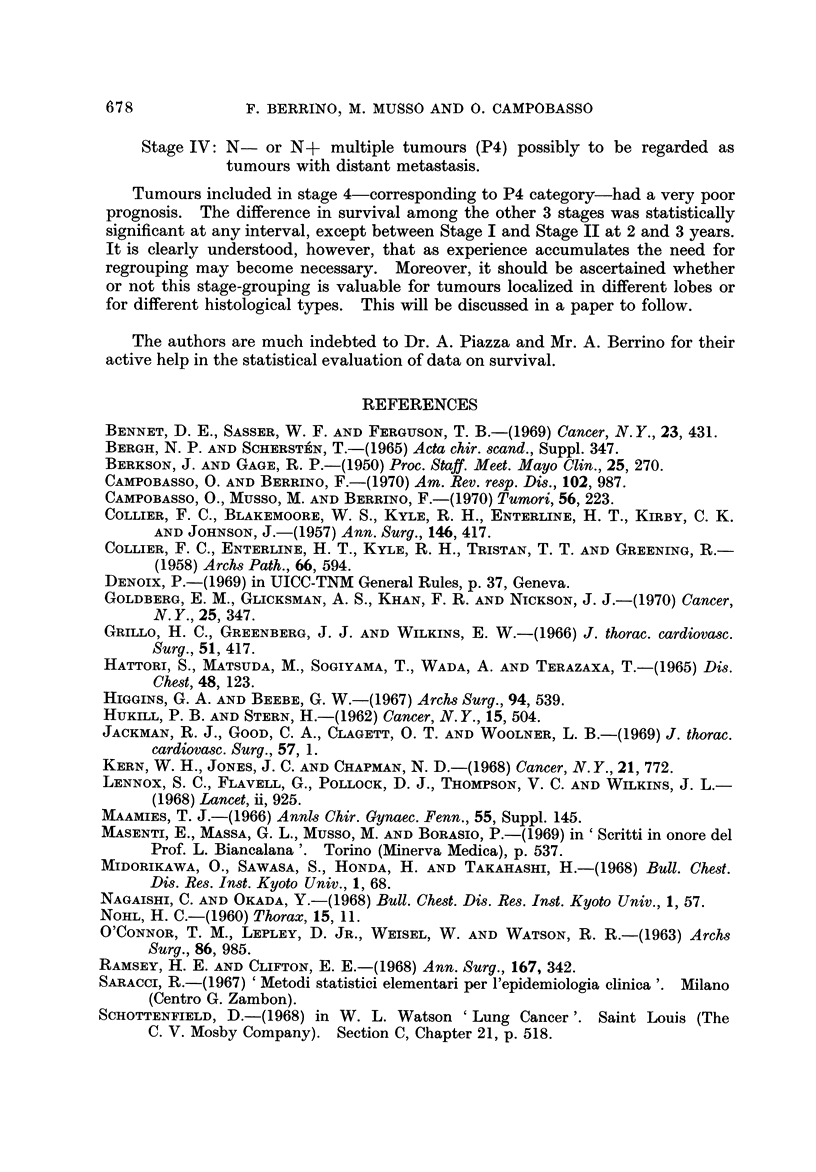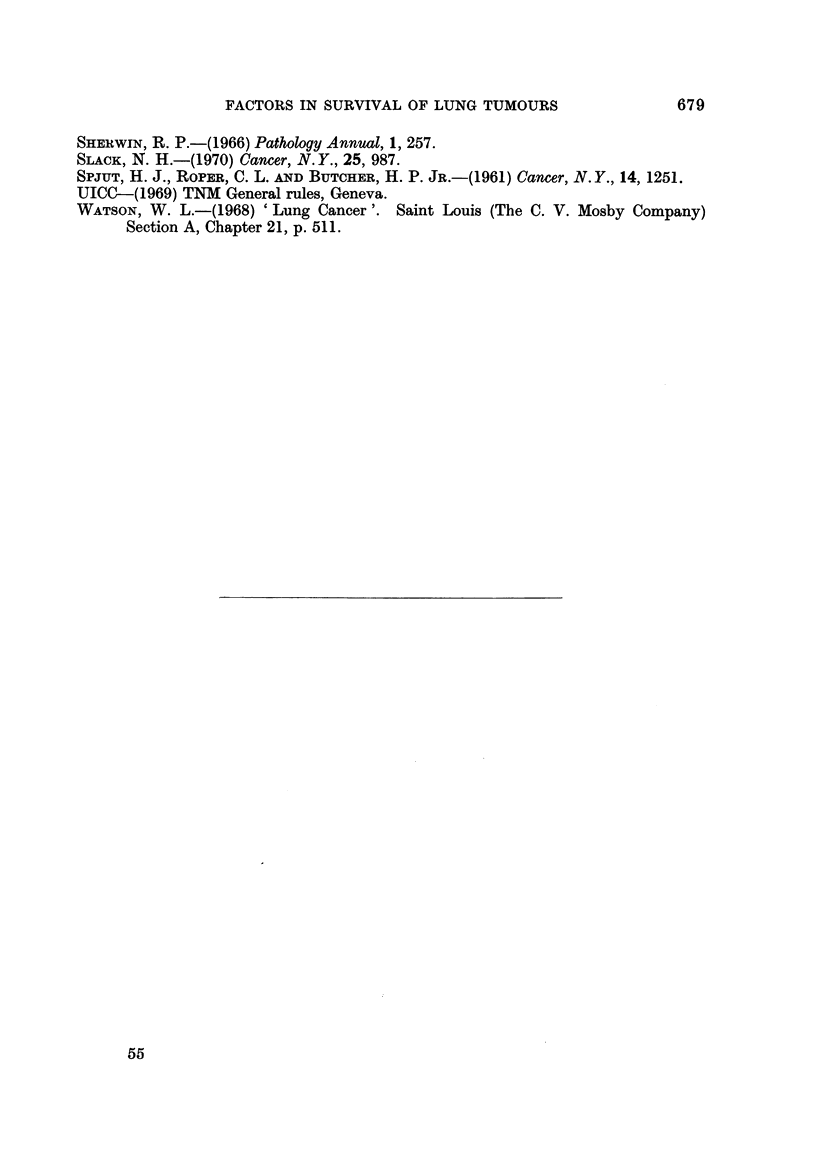# Pathological Factors in Survival of Lung Tumours: Local Extent, Size, and Nodal Involvement

**DOI:** 10.1038/bjc.1971.82

**Published:** 1971-12

**Authors:** F. Berrino, M. Musso, O. Campobasso

## Abstract

The pathological features, particularly local extent, size, and nodal involvement, of 405 surgical specimens of human lung carcinomas were studied. A direct relationship was found between local extent and size of the tumour and between local extent and the incidence of lymph node metastasis, but not between tumour size and the incidence of lymph node metastasis. The survival rates in the 405 tumours were calculated with the actuarial method in relation to the 3 pathological factors: local extent, lymph node metastasis and tumour size showed a predictive value in prognosis of lung tumours. Their prognostic value, however, was much more meaningful when the three pathological factors were considered in relation to each other. As a matter of fact, the size of the tumour showed no predictive value when lymph node metastasis was present. On the ground of the mutual influence of the 3 factors in affecting prognosis a pathological stage-grouping of lung tumours has been suggested.


### PATHOLOGICAL FACTORS IN SURVIVAL OF LUNG TUMOURS: LOCAL EXTENT, SIZE, AND NODAL INVOLVEMENT

F. BERRINO, M. MUSSO AND O. CAMPOBASSO*

*From the Institute of Morbid Anatomy and the Thoracic Surgery Centre, University of Turin, Turin, Italy*

Received for publication April 28, 1971

**SUMMARY.—The pathological features, particularly local extent, size, and nodal involvement, of 405 surgical specimens of human lung carcinomas were studied. A direct relationship was found between local extent and size of the tumour and between local extent and the incidence of lymph node metastasis, but not between tumour size and the incidence of lymph node metastasis. The survival rates in the 405 tumours were calculated with the actuarial method in relation to the 3 pathological factors: local extent, lymph node metastasis and tumour size showed a predictive value in prognosis of lung tumours. Their prognostic value, however, was much more meaningful when the three pathological factors were considered in relation to each other. As a matter of fact, the size of the tumour showed no predictive value when lymph node metastasis was present. On the ground of the mutual influence of the 3 factors in affecting prognosis a pathological stage-grouping of lung tumours has been suggested.**

ATTEMPTS to find clinical as well as pathological factors bearing on the prognosis of lung tumours have been for the most part unrewarding (Watson, 1968; Bennet *et al.*, 1969). General agreement has been reached on the predictive value of nodal involvement, which lowers significantly the survival rate (Nohl, 1960; Hukill and Stern, 1962; Bergh and Sherstén, 1965; Nagaishi and Okada, 1968; Goldberg *et al.*, 1970). The value of other pathological factors like size of the tumour, pleural and/or vascular involvement, histological type, site and/or location of the tumour (Collier *et al.*, 1958; Spjut *et al.*, 1961; Maamies, 1966; Schottenfield, 1968; Jackman *et al.*, 1969; Bennet *et al.*, 1969; Slack, 1970) has been emphasized by some authors and denied by others.

Higgins and Beebe (1967) found that only 4 or 5 out of 40 both clinical and pathological factors examined carried independent information predictive of cancer-free survival at 36 or 60 months. Midorikawa *et al.* (1968) found that it was difficult to predict the prognosis of resected lung tumours on the basis of pathological examination.

This disagreement can be in part explained by the limitations of routine pathological examination (Sherwin, 1966) and more generally with the rather rough definition of some of the studied factors. A second point which seems worthwhile considering is that most of these factors have generally been taken into account one at a time. Some factors may likely play a different role in affecting prognosis whether they are or are not related to other factors.

* Correspondence to: Dr O. Campobasso, Instituto di Anatomia Patologica, Via Santena 7, 10126, Torino, Italy.

In a recent paper (Campobasso *et al.*, 1970) we tried to give a clear-cut definition of local extent of lung tumours taking into account the site of the tumour, the involvement of main blood vessels, and the spread to contiguous or neighbouring structures such as visceral or parietal pleura, main bronchus, chest wall, etc. (excluding lymph nodes). Tumours were subdivided into 4 categories of local extent, denoted by the symbol P—degrees of histopathological extent, according to the term suggested by the Union Internationale Contre le Cancer (UICC, 1969). The main aim of this classification was to supply a basis for an accurate recording of the extent of lung tumours.

It is the purpose of this present paper to evaluate the relationship of local extent with 2 other factors in survival, *i.e.*, size of the tumour and nodal involvement and to establish the significance of these 3 pathological factors, independently and when considered in relation to each other. The histological type will not be taken into account; it will be discussed in a future paper.

### MATERIAL AND METHODS

Five hundred and forty-six surgical specimens of lung carcinomas, obtained from the Thoracic Surgery Centre of the University of Turin, up to the December 31, 1965, have been studied. All the available data for these patients have been reported on marginal punch cards. One hundred and forty-one cases were excluded from the series: 33 for lack of information about survival; 30 because the pathological recording was not sufficient for an accurate classification; 7 because the bronchopulmonary origin of the tumour was doubtful; 71 because of post-operative death (1 month). As previously stated histological type has not been taken into account in this study. However, all the tumours included in the present series were frank invasive carcinomas. Carcinoids, mucous-gland tumours, bronchial papillomata, lymphomas and other rare tumours were excluded.

The present series includes 405 cases for which sufficient clinical and pathological data were available. For the present study the following data have been taken into account:

(a) the category of local extent (P) (Campobasso *et al.*, 1970). Lung tumours have been subdivided into 4 categories of local extent as follows: P1, central or peripheral tumours confined to the lung; P2, tumours involving the main bronchus or the visceral pleura, excluding the pleura lining the fissures; P3, tumours with spread to mediastinal soft tissues and/or other mediastinal structures (excluding lymph nodes) such as pericardium and main blood vessels, or to parietal pleura and chest wall including diaphragm; P4, tumours with 2 or more separate neoplastic masses in the same lobe or in different lobes of the same lung.

(b) the size of the tumour. Tumours have been subdivided into tumours up to 4 cm. in diameter and tumours larger than 4 cm. in diameter. This arbitrary limit has been chosen because it has been adopted by other authors (O'Connor *et al.*, 1963; Bennet *et al.*, 1969; Jackman *et al.*, 1969). The size was taken into account in 379 tumours only, excluding multiple tumours (P4 tumours, 26 cases) where it would have been difficult and perhaps useless to measure the size of the whole neoplastic tissue.

(c) the involvement of regional lymph nodes. This has been checked macroscopically and microscopically. One to 6 lymph nodes have been examined for each case under the microscope, generally those which were macroscopically more suspicious; moreover only in a certain number of cases data on the exact site of the resected lymph nodes were available. So the presence of lymph node metastasis (indicated with N+) signifies that 1 or more lymph nodes, regardless of their site and number, were involved by the tumour, while absence of metastasis (indicated with N—) signifies that in none of the examined lymph nodes were neoplastic cells present.

(d) the data on the survival. At the Thoracic Surgery Centre of the University of Turin, people from every region of Italy are admitted. This made it impossible to re-examine directly all the patients who had been operated on, months or years before. In many cases information on the course of the disease was obtained by letters from patients themselves or from their relatives, so that it has not been possible to take into account the possible presence of recurrences at the moment of the follow-up or the causes of death, as well as the data on post-operative therapy. Consequently, people alive at the moment of the last control have been regarded as survivors irrespective of the presence of recurrences. Dead people were regarded as being so due to their lung tumours. However, when known, the cause of death was clearly referable to the tumour. Of the 405 patients included in the present series, 404 were followed-up for more than 2 years, 369 for more than 3 years, and 298 for 5 years or more.

The survival rates have been calculated by the actuarial method of Berkson and Gage (1950). The 95% confidence limits of survival rates and the statistical significance of the observed differences have been calculated applying the formula of Greenwood quoted by Denoix (1969); a further control of the statistical significance has been made with Fischer's exact probability method comparing the absolute values instead of the corresponding percentages. The significance level has been chosen at 5% ($P < 0{\cdot}05$).

### RESULTS

Out of 405 tumours, 223 (55·1%) were classified as P1, 111 (27·4%) as P2, 45 (11·1%) as P3 and 26 (6·4%) as P4. The incidence of lymph node metastasis was 30·3% in the whole series, ranging from 30·6% to 69·2% in the 4 P categories (Table I). The percentage of tumours up to 4 cm. in diameter ranged from 43% in P1 category to 16·2% in P2 category. The difference in the incidence of lymph node metastasis between P1 and P2 on one side and P3 and P4 on the other side was statistically significant. The incidence of small tumours in P1 was significantly lower than in P2 and P3. There was practically no difference in the incidence of lymph node metastasis between small and large tumours (Table II).

In the whole series the survival rates at 2 years, 3 years and 5 years were 36·5%, 30·7% and 23·5% respectively (Table III).

Table III reports also the survival rates according to P. These rates decreased inversely to the local extent and were extremely low in P4 tumours; the difference in survival between P1 and any other P category was statistically significant; the survival rate was nearly the same at any time in P2 and P3 tumours.

Tables IV and V show the survival rates according to nodal involvement and size of the tumour respectively. The differences in survival were statistically significant at 2, 3 and 5 years for both N— versus N+ tumours ($P < 0{\cdot}01$) and small versus large tumours ($P < 0{\cdot}001$).

The survival patterns by P category and lymph node metastasis, and by P category and size, are shown in Fig. 1 and 2 respectively.

TABLE I.—*Relationship Between P and N and P and Size*

| P category | No. | Nodal involvement: N − No. | N − % | N + No. | N + % | Tumour size: ⩽ 4 cm. No. | ⩽ 4 cm. % | > 4 cm. No. | > 4 cm. % |
|---|---|---|---|---|---|---|---|---|---|
| P1 | 223 | 143 | 64·1 | 80 | 35·9 | 96 | 43·0 | 127 | 57·0 |
| P2 | 111 | 77 | 69·4 | 34 | 30·6 | 18 | 16·2 | 93 | 83·8 |
| P3 | 45 | 18 | 40·0 | 27 | 60·0 | 11 | 24·5 | 34 | 75·5 |
| P4 | 26 | 8 | 30·8 | 18 | 69·2 | * | — | * | — |

* Size has not been taken into account in P4 tumours.

TABLE II.—*Relationship Between Size and N (Excluding P4 Tumours)*

| Tumour size | No. | Nodal involvement: N − No. | N − % | N + No. | N + % |
|---|---|---|---|---|---|
| ⩽4 cm. | 125 | 81 | 64·8 | 44 | 35·2 |
| >4 cm. | 254 | 157 | 61·8 | 97 | 38·2 |

TABLE III.—*Survival Rates in 405 Resected Lung Tumours According to P Category*

| P category | No. | Survival rate at 2 years % | 3 years % | 5 years % |
|---|---|---|---|---|
| P1 | 223 | 49·26±7 | 42·70±7 | 33·89±7 |
| P2 | 111 | 26·13±8 | 19·59±8 | 12·74±6 |
| P3 | 45 | 17·78±11 | 15·56±11 | 11·11±9 |
| P4 | 26 | 3·85±8 | — | — |
| Total | 405 | 36·53±5 | 30·71±4 | 27·47±4 |

TABLE IV.—*Survival Rates in 405 Resected Lung Tumours According to Nodal Involvement*

| Nodal involvement | No. | Survival rate at 2 years % | 3 years % | 5 years % |
|---|---|---|---|---|
| N− | 246 | 46·34±6 | 38·37±6 | 29·32±6 |
| N+ | 156 | 21·21±6 | 18·52±6 | 13·84±6 |

TABLE V.—*Survival Rates in 379 Resected Lung Tumours According to Tumour Size (Excluding P4 Tumours)*

| Tumour size | No. | Survival rate at 2 years % | 3 years % | 5 years % |
|---|---|---|---|---|
| ⩽4 cm. | 125 | 53·60±9 | 46·90±9 | 38·20±9 |
| >4 cm. | 254 | 31·42±6 | 25·71±6 | 18·36±5 |

TABLE VI.—*Survival Rates in 379 Resected Lung Tumours According to N and Size**

| Nodal involvement | Tumour size (cm.) | No. | Survival rate at 2 years % | 3 years % | 5 years % |
|---|---|---|---|---|---|
| N− | ≤4 | 81 | 65·43±11 | 56·54±11 | 48·07±11 |
| | >4 | 157 | 38·85±8 | 30·95±7 | 21·12±7 |
| N+ | ≤4 | 44 | 31·82±14 | 29·27±14 | 19·32±13 |
| | >4 | 97 | 19·28±8 | 17·14±8 | 13·69±7 |

* P4 tumours have not been taken into account.

FIG. 1.—Survival by P category in N— (a) and N+ (b) tumours.

Table VI shows the survival rates according to size in N— and N+ tumours, regardless of P category. The difference in survival between small and large tumours at 2 years, 3 years, and 5 years was statistically significant ($P < 0{\cdot}01$ at any interval) in N— tumours only. The difference was much less evident and statistically not significant at any interval in N+ tumours.

Table VII shows the survival rates in the 405 resected lung tumours subdivided by category of local extent (P) and according to nodal involvement (N) and size.

TABLE VII.—*Survival Rates in 405 Resected Lung Tumours According to P, N and Size*

| P category | Nodal involvement | Size (cm.) | No. | Survival rate at 2 years % | 3 years % | 5 years % |
|---|---|---|---|---|---|---|
| P1 | N− | ⩽4 | 59 | 66·10±12 | 59·05±13 | 55·11±13 |
| | | >4 | 84 | 53·57±11 | 45·05±11 | 30·92±11 |
| | N+ | ⩽4 | 37 | 35·16±16 | 32·15±15 | 24·13±15 |
| | | >4 | 43 | 29·66±15 | 24·72±13 | 19·28±12 |
| P2 | N− | ⩽4 | 16 | 75·00±22 | 56·25±25 | 31·25±23 |
| | | >4 | 61 | 21·31±10 | 14·21±9 | 8·12±7 |
| | N+ | ⩽4 | 2 | — | — | — |
| | | >4 | 32 | 12·50±12 | 12·50±12 | 12·50±12 |
| P3 | N− | ⩽4 | 6 | 33·33±38 | 33·33±38 | 33·33±38 |
| | | >4 | 12 | 25·00±25 | 16·67±22 | 16·67±22 |
| | N+ | ⩽4 | 5 | 20·00±36 | 20·00±36 | — |
| | | >4 | 22 | 9·09±12 | 9·09±12 | 4·55±9 |
| P4 | N− | * | 8 | — | — | — |
| | N+ | * | 18 | 5·56±11 | — | — |

* Size has not been taken into account in P4 tumours.

FIG. 2.—Survival by P category in tumours up to 4 cm. in diameter (a) and larger than 4 cm. (b).

P1 N— tumours up to 4 cm. in diameter yielded the highest 5 years survival rate (55·1%). P1 N— tumours larger than 4 cm. and both P2 N— and P3 N— tumours up to 4 cm. showed intermediate figures (more than 30% alive at 5 years). P2 N— and P3 N— large tumours, as well as N+ tumours yielded a rather poor survival (less than 25% alive at 5 years). In P4 tumours the size was not taken into account and the survival was very poor for both N— and N+ lesions.

### DISCUSSION

Most of the tumours in the present series were confined to the lung (223 = 55·1%) and/or had not spread to lymph nodes (246 = 60·7%). This is not surprising in a surgical series and it is clearly understood that these figures may not be related to lung tumours in general. At the Thoracic Surgery Centre of Turin the resectability rate for lung cancer was found to be 29% (Masenti *et al.*, 1969). As the resectability is in the main directly related to the spread of the tumour, this means that only a small proportion of the lung tumours seen at the Thoracic Surgery Centre of Turin up to December 31, 1965 were confined to the lung and had not spread to lymph nodes when first diagnosed.

The 5 years survival rate in the whole series of 405 resected cases was 23·37%, similar to that reported by many authors (Collier *et al.*, 1957; Bergh and Scherstén, 1965; Maamies, 1966; Watson, 1968; Kern *et al.*, 1968; Slack, 1970).

All the 3 pathological factors taken into account, when examined one at a time (Tables III, IV and V), carried significant information predictive of post-operative survival. Apparently the size of the tumour was the most important factor in survival as at 5 years, when examined independently from the other factors, small tumours yielded the highest survival rate (38·20%), following tumours confined to the lung (P1, 33·89%), and tumours without nodal involvement (N—, 29·32%). When their relationships were considered, however, a complicated but meaningful pattern of associations and mutual influences was detected among these factors. The somewhat different meaning and importance of the various factors considered in conjunction with one another need some comment.

As for the local extent there was a direct relationship between P category and both the incidence of node metastasis and the size of the tumour (Table I). The lower incidence of node metastasis in more locally extended tumours is in agreement with the findings of Nohl (1960), who classified lung tumours in 3 categories of local extent, A, B, and C, roughly corresponding to our P1 to P3 categories. In the present series however, the predictive value of local extent was rather independent of the incidence of node metastasis in the different P categories; in fact both N— and N+ tumours (Fig. 1a, b) showed a survival pattern by P category very similar to that of the whole series (Table III), showing a marked difference between P1 and P2 and approximately the same survival rates in P2 and P3 categories. The influence of lymph node metastasis was only in that the difference between P1 and the other P categories as a whole was statistically significant at any interval in N— tumours ($P < 0{\cdot}01$) but only at 2 years and 3 years in N+ tumours ($P < 0{\cdot}05$). In P2 category the incidence of lymph node metastasis was somewhat lower than in P1. The very high percentage of large lesions (83·8%, Table I) among P2 tumours may partly account for their poor prognosis. In small tumours indeed there was some overlapping in survival between P1 and P2 categories at 2 and 3 years and a difference—though not statistically significant

—between P2 and P3 tumours at 2 years (Fig. 2a). In large tumours (Fig. 2b) the survival pattern by P category was quite consistent with that of the whole series (Table III). Neither the nodal involvement nor the tumour size, however, were useful for a clear cut difference in prognosis between P2 and P3 tumours. The lack of difference between these 2 categories is not in agreement with the findings of Nohl (1960) and of Bergh and Scherstén (1965). The latter authors found a marked difference between their A and B groups and C group of tumours. On the other hand, in oat cell carcinomas Lennox *et al.* (1968) found the same survival rate in tumours involving the visceral pleura and the chest wall. These discrepancies may partly be due to the different criteria in classifying tumours as well as to the different incidence of pathological factors in the various series. One must consider that the involvement of some structures is probably more dangerous than the involvement of other structures. Bergh and Scherstén (1965) showed that the perinodal growth (*i.e.* the invasion of mediastinal soft tissues) in cases with lymph node metastasis bore very badly on prognosis. On the other hand, extended resection for tumours locally involving the chest wall has been stressed by some surgeons as very valuable for cure, when lymph node metastasis is absent (Grillo *et al.*, 1966; Ramsey and Clifton, 1968). In the present series 37 out of 45 patients included in P3 category had a 5 year follow-up; of 5 survivors, 4 had been included in P3 category as the tumours had involved the thoracic wall; none had lymph node metastasis. Moreover, Bennet *et al.* (1969) pointed out that as far as pleural invasion was concerned, only pleura implants or permeation of subpleural lymphatics were adverse factors in prognosis. So the distinction between P2 and P3 tumours which proved very useful in reporting the pathological features of lung tumours, seems to be of little predictive value in survival.

P4 tumours had a very poor prognosis; no patient with multiple tumours survived up to 3 years and only 1 out of 26 survived for 2 years. It has been postulated (Campobasso *et al.*, 1970) that the reason why these tumours have such a poor prognosis is that in these cases 1 neoplastic mass is the primary lung tumour and the other mass or masses are distant lung metastases of the primary lung tumour through the blood stream.

Nodal involvement has been regarded as one of the most important factors in survival. The data of the present series are in agreement with those of other authors. The presence of lymph node metastasis affected markedly the predictive value of the other factors, except for the P4 category, in which distant metastasis were probably present, and for P2 large tumours, in which size accounted for the poor prognosis (Table VII). In any case, in N+ tumours there was no statistically significant difference in survival at 5 years amongst the 4 P categories (Fig. 1b). Moreover, nodal involvement clearly affected the predictive value of tumour size, as the survival experience of small tumours was significantly better than that of large ones provided lymph node metastasis was absent (Table VI).

The size of the tumour showed no relation to the incidence of nodal metastasis (Table II). As has been pointed out elsewhere (Campobasso and Berrino 1970), this makes it difficult to regard small tumours as early lung tumours as some authors do (Hattori *et al.*, 1965; Nagaishi and Okada, 1968). There is no exhaustive mention in the literature of the relationship between size and nodal involvement in lung tumours, and generally the value of tumour size has not been evaluated in relation to lymph node metastasis. This may well explain why the predictive value of tumour size has been reported with contradictory results. O'Connor

*et al.* (1963), Nagaishi and Okada (1968) and Jackman *et al.* (1969) regarded small tumours as candidates for surgery and cure. Hukill and Stern (1962) denied that size had a predictive value in prognosis. Bennet *et al.* (1969) found that size had only little value mainly because " small size does not necessarily denote a biologically early lesion "; 3 out of 7 of their small tumours had positive lymph nodes at the time of resection. Though the author has not fully got to the bottom of this point, it is clear from Table 6 of the recent paper by Slack (1970) that 5 years survival rates decreased significantly with the increase of tumour size only when nodal involvement was absent. In the present series tumours up to 4 cm. in diameter as a whole had a much better prognosis than tumours larger than 4 cm. (Table V) possibly because the incidence of nodal involvement in the total series was rather slow. It is clear, however, that when lymph node metastasis is present at the time of resection, tumour size does not bear significantly on prognosis of lung tumours.

TABLE VIII.—*Pathological Stage-grouping of Lung Tumours*

| Stage | No. | P | Size (cm.) | N | Other factors | Survival rate at 2 years % | 3 years % | 5 years % |
|---|---|---|---|---|---|---|---|---|
| I | 59 | P1 | ⩽4 | N− | — | 66·1±12 | 59·0±13 | 55·1±13 |
| II | 106 | P1 | >4 | N− | — | 55·7±10 | 46·1±10 | 31·1±9 |
| | | P2 | ⩽4 | N− | — | | | |
| | | P3 | ⩽4 | N− | — | | | |
| III | 214 | P2 | >4 | N− | — | 22·8±6 | 18·8±5 | 13·7±5 |
| | | P3 | >4 | N− | — | | | |
| | | P1 | any size | N+ | — | | | |
| | | P2 | any size | N+ | — | | | |
| | | P3 | any size | N+ | — | | | |
| IV | 26 | P4 | any size | N− / N+ | distant metastasis (?) | 3·8±8 | — | — |

The outcome of this present investigation has suggested that local extent, nodal involvement and size are pathological factors of predictive value in prognosis of lung tumours. However, they should not be taken into account one at a time. Their predictive value, indeed, is much more meaningful when these factors are correlated with each other, as the predictive value of one factor may be cancelled by the association with another factor. This has been clearly demonstrated, in the present series, for the tumour size. Correlating these factors with each other in evaluating their influence on survival, is imperative, therefore, and may be useful for a stage-grouping. On the ground of their significance and relationship in the present series, the following pathological stage-grouping of lung tumours may be tentatively suggested (Table VIII):

Stage I: N— tumours confined to the lung (P1), up to 4 cm. in diameter.

Stage II: N— tumours confined to the lung (P1) but larger than 4 cm. and N— tumours spread to contiguous or neighbouring structures (P2 and P3), up to 4 cm. in diameter.

Stage III: N— tumours spread to contiguous or neighbouring structures (P2, P3) larger than 4 cm.; N+ tumours of any size, confined to the lung or spread to contiguous or neighbouring structures (P1, P2, P3).

Stage IV: N— or N+ multiple tumours (P4) possibly to be regarded as tumours with distant metastasis.

Tumours included in stage 4—corresponding to P4 category—had a very poor prognosis. The difference in survival among the other 3 stages was statistically significant at any interval, except between Stage I and Stage II at 2 and 3 years. It is clearly understood, however, that as experience accumulates the need for regrouping may become necessary. Moreover, it should be ascertained whether or not this stage-grouping is valuable for tumours localized in different lobes or for different histological types. This will be discussed in a paper to follow.

The authors are much indebted to Dr. A. Piazza and Mr. A. Berrino for their active help in the statistical evaluation of data on survival.

### REFERENCES

Bennet, D. E., Sasser, W. F. and Ferguson, T. B.—(1969) *Cancer, N.Y.*, **23**, 431.
Bergh, N. P. and Scherstén, T.—(1965) *Acta chir. scand.*, Suppl. 347.
Berkson, J. and Gage, R. P.—(1950) *Proc. Staff. Meet. Mayo Clin.*, **25**, 270.
Campobasso, O. and Berrino, F.—(1970) *Am. Rev. resp. Dis.*, **102**, 987.
Campobasso, O., Musso, M. and Berrino, F.—(1970) *Tumori*, **56**, 223.
Collier, F. C., Blakemoore, W. S., Kyle, R. H., Enterline, H. T., Kirby, C. K. and Johnson, J.—(1957) *Ann. Surg.*, **146**, 417.
Collier, F. C., Enterline, H. T., Kyle, R. H., Tristan, T. T. and Greening, R.—(1958) *Archs Path.*, **66**, 594.
Denoix, P.—(1969) in UICC-TNM General Rules, p. 37, Geneva.
Goldberg, E. M., Glicksman, A. S., Khan, F. R. and Nickson, J. J.—(1970) *Cancer, N.Y.*, **25**, 347.
Grillo, H. C., Greenberg, J. J. and Wilkins, E. W.—(1966) *J. thorac. cardiovasc. Surg.*, **51**, 417.
Hattori, S., Matsuda, M., Sogiyama, T., Wada, A. and Terazaxa, T.—(1965) *Dis. Chest*, **48**, 123.
Higgins, G. A. and Beebe, G. W.—(1967) *Archs Surg.*, **94**, 539.
Hukill, P. B. and Stern, H.—(1962) *Cancer, N.Y.*, **15**, 504.
Jackman, R. J., Good, C. A., Clagett, O. T. and Woolner, L. B.—(1969) *J. thorac. cardiovasc. Surg.*, **57**, 1.
Kern, W. H., Jones, J. C. and Chapman, N. D.—(1968) *Cancer, N.Y.*, **21**, 772.
Lennox, S. C., Flavell, G., Pollock, D. J., Thompson, V. C. and Wilkins, J. L.—(1968) *Lancet*, ii, 925.
Maamies, T. J.—(1966) *Annls Chir. Gynaec. Fenn.*, **55**, Suppl. 145.
Masenti, E., Massa, G. L., Musso, M. and Borasio, P.—(1969) in ‘ Scritti in onore del Prof. L. Biancalana ’. Torino (Minerva Medica), p. 537.
Midorikawa, O., Sawasa, S., Honda, H. and Takahashi, H.—(1968) *Bull. Chest. Dis. Res. Inst. Kyoto Univ.*, **1**, 68.
Nagaishi, C. and Okada, Y.—(1968) *Bull. Chest. Dis. Res. Inst. Kyoto Univ.*, **1**, 57.
Nohl, H. C.—(1960) *Thorax*, **15**, 11.
O'Connor, T. M., Lepley, D. Jr., Weisel, W. and Watson, R. R.—(1963) *Archs Surg.*, **86**, 985.
Ramsey, H. E. and Clifton, E. E.—(1968) *Ann. Surg.*, **167**, 342.
Saracci, R.—(1967) ‘ Metodi statistici elementari per l'epidemiologia clinica ’. Milano (Centro G. Zambon).
Schottenfield, D.—(1968) in W. L. Watson ‘ Lung Cancer ’. Saint Louis (The C. V. Mosby Company). Section C, Chapter 21, p. 518.

SHERWIN, R. P.—(1966) *Pathology Annual*, **1**, 257.
SLACK, N. H.—(1970) *Cancer, N.Y.*, **25**, 987.
SPJUT, H. J., ROPER, C. L. AND BUTCHER, H. P. JR.—(1961) *Cancer, N.Y.*, **14**, 1251.
UICC—(1969) TNM General rules, Geneva.
WATSON, W. L.—(1968) 'Lung Cancer'. Saint Louis (The C. V. Mosby Company) Section A, Chapter 21, p. 511.